# Carbon Fiber/Polyether Ether Ketone (CF/PEEK) Implants Allow for More Effective Radiation in Long Bones

**DOI:** 10.3390/ma13071754

**Published:** 2020-04-09

**Authors:** Christoph J. Laux, Christina Villefort, Stefanie Ehrbar, Lotte Wilke, Matthias Guckenberger, Daniel A. Müller

**Affiliations:** 1 Department of Orthopaedics, Balgrist University Hospital, University of Zurich, Forchstrasse 340, 8008 Zurich, Switzerland; 2 Department of Radiation Oncology, University Hospital Zurich, University of Zurich, Rämistrasse 100, 8091 Zurich, Switzerland

**Keywords:** bone metastasis, orthopedic oncology, carbon fiber polyether ether ketone, radiation therapy, imaging artefact, dose deviation

## Abstract

Background: Metallic implants show dose-modulating effects in radiotherapy and complicate its computed tomography (CT)-based planning. Dose deviations might not only affect the surrounding tissues due to backscattering and inadvertent dose increase but might also compromise the therapeutic effect to the target lesion due to beam attenuation. Later on, follow-up imaging is often obscured by metallic artefacts. *Purposes:* This study investigates the dosimetric impact of titanium and radiolucent carbon fiber/polyether ether ketone (CF/PEEK) implants during adjuvant radiation therapy in long bones. (1) Does the use of CF/PEEK implants allow for a more homogenous application of radiation? (2) Is the dose delivery to the target volume more efficient when using CF/PEEK implants? (3) Do CF/PEEK implants facilitate CT-based radiation therapy planning? *Materials and methods:* After CT-based planning, bone models of six ovine femora were irradiated within a water phantom in two immersion depths to simulate different soft-tissue envelopes. Plates and intramedullary nails of both titanium and CF/PEEK were investigated. Radiation dosage and distribution patterns were mapped using dosimetry films. *Results:* First, the planned implant-related beam attenuation was lower for the CF/PEEK plate (1% vs. 5%) and the CF/PEEK nail (2% vs. 9%) than for corresponding titanium implants. Secondly, the effective decrease of radiation dosage behind the implants was noticeably smaller when using CF/PEEK implants. The radiation dose was not significantly affected by the amount of surrounding soft tissues. A significant imaging artefact reduction was seen in all CF/PEEK models. *Conclusion:* CF/PEEK implants lead to a more reliable and more effective delivery of radiation dose to an osseous target volume. With regard to radiation therapy, the use of CF/PEEK implants appears to be particularly beneficial for intramedullary nails.

## 1. Introduction

In orthopedic oncology, a surgical stabilization of the affected bone is often required after thorough assessment of the clinical context and prognostic factors [1]. The individual fracture risk of long bones metastases can be calculated using the Mirels’ score [2]. Radiation therapy is an important oncological adjunct in the management of these patients, especially when encountering multiple or osteolytic lesions. However, metallic implants show a dose-modulating effect in radiotherapy and complicate its CT-based planning [3,4]. During radiation therapy, intra or extramedullary implants might not only affect the surrounding tissue due to backscattering and inadvertent dose increase but might also compromise the therapeutic effect to the target lesion due to beam attenuation. Later on, follow-up imaging and the diagnosis of local recurrences is often obscured by metallic artefacts [5]. This is why radiolucent implant materials, in particular, for the use in orthopedic tumor patients, are increasingly being recognized [6,7]. While carbon fiber/polyether ether ketone (CF/PEEK) implants have already been examined biomechanically, data on their radiophysical properties are scarce [8,9,10,11].

The only study analyzing the beam attenuation conditioned by CF/PEEK implants in a solid water phantom was published by Nevelsky et al. [3]. CF/PEEK pedicle screws featured no backscatter effect and a minimal dose attenuation when compared to titanium pedicle screws. The maximum dose of backscattering to adjacent tissues was 10% in titanium screws, whereas CF/PEEK screws did not show a backscattering effect at all. Additionally, the radiation beam was attenuated by 30% when using titanium screws, whereas CF/PEEK screws showed only minimal dose alteration with a calculated attenuation of 5%. Nevertheless, the amount of dose modulation within the bone and the implant-bone interface has not yet been quantified.

This experimental study therefore aims to investigate the potential benefits of CF/PEEK implants during planning and administration of adjuvant radiation therapy to the extremities and to quantify the disturbing influence of orthopedic implants on radiation dosage and distribution patterns. (1) Does the use of CF/PEEK implants allow for a more homogenous and therefore more predictable application of radiation? (2) Is the dose delivery to the target volume more efficient when using CF/PEEK implants? (3) Do CF/PEEK implants facilitate CT-based radiation therapy planning? For this purpose, we deployed an ovine bone model within a water phantom and compared different implant designs and materials.

## 2. Material and Methods

### 2.1. Experimental Setup

**Figure 1:**
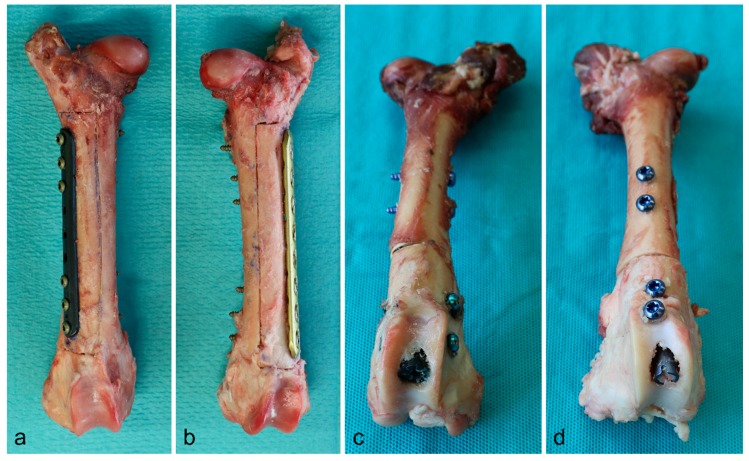
Bone preparation with plates (**a**: CF/PEEK, **b**: titanium) and intramedullary nails (**c**: CF/PEEK, **d**: titanium) in situ.

Since water strongly resembles the radiodensity of the soft tissues, we used a water phantom for the examination of radiation variances around different titanium and carbon/PEEK implants at the extremities. We decided to use ovine femora with respect to the well transferable implant dimensions of human osteosynthesis implants. Thereby, we could assure that common types of fixation as used in clinical practice were simulated. We implanted intramedullary nails (CarboFix Piccolo Proximal Humerus Nail 150 mm × 8 mm, DePuy Synthes Expert Proximal Humeral Nail 150 mm × 7 mm) in one pair of bones, and plates (CarboFix Piccolo One Third Tubular 98 mm/9 holes, DePuy Synthes 3.5 mm Locking Compression Plate 111 mm/8 holes) in a second pair of bones. We chose implants with the same length of the screw-free interval after screw fixation using three screws on either plate side for the simulation of an equally large tumorous lesion. We exclusively used titanium screws for implant fixation in every bone. A third pair of bones was used for implant-free reference measurements. The bone preparation is illustrated in Figure 1.

**Figure 2:**
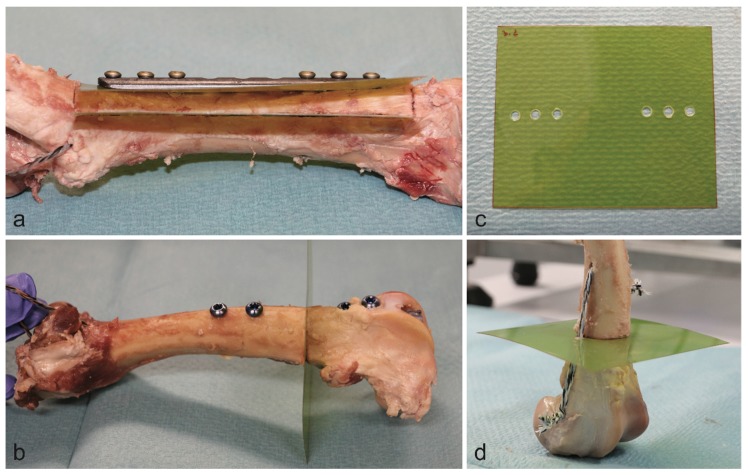
Position of dosimetry films in the plate model (**a**) and the intramedullary nail model (**b**). Dosimetry film with punch holes for the plate model (**c**). Reference bone of the nail model (**d**).

The radiation dosage was mapped with dosimetry films (Gafchromic EBT 2, Ashland Inc., Covington, KY, USA) parallel to the plates and orthogonal to the intramedullary nails. Holes with appropriate diameters were punched into dosimetry films to fit around the screws or the intramedullary nails, respectively. In the plate models, one film was placed between the plate and the bone. A second film was placed within the bone and parallel to the plate after sawing out a box with a thickness of 10 mm. For the reference bone of the nail model, a supplementary suture fixation was necessary to hold both ends of the bone together. For this reason, the dosimetry film was pierced laterally. The preparation and the respective orientation of the dosimetry films is shown in Figure 2 for all types of implants.

**Figure 3:**
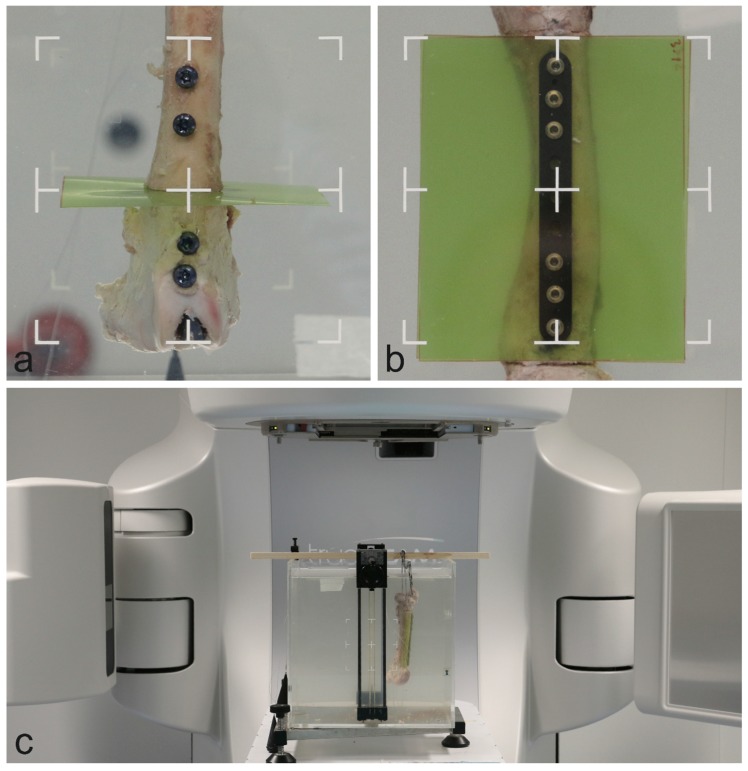
Specimen orientation within the water basin in the intramedullary nail model (**a**) and the plate model (**b**). Full setup within the linear accelerator (**c**).

The bones were placed within a cubic water basin. For the intramedullary nail setup, the radiation beam was oriented parallel to the dosimetry film and centered on the bone (Figure 3). The radiation beam was centered on the bone and between the two most central screws in the plate model. Each model was deployed with immersion depths of 5 and 10 cm for simulation of the upper and lower extremities, respectively.

### 2.2. Measurements

**Figure 4:**
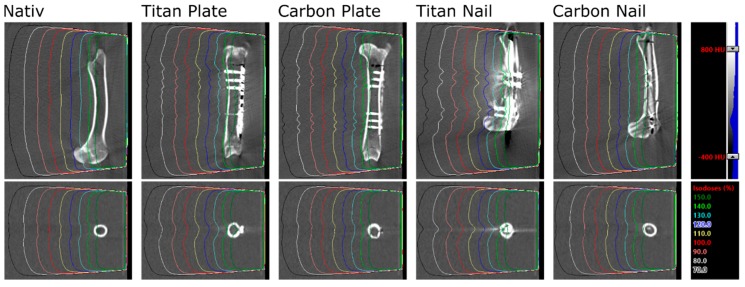
CT images and calculated dose distributions for all bones and implants at 5 cm immersion depth. Densities from −400 to 800 HU are shown in grey scale; isodose levels of a 6 MV, 20 × 20 cm^2^ field are shown in color.

For each bone and setup, a computed tomography (CT) scan of the water phantom including the bone was obtained (SOMATOM Definition AS Open scanner, Siemens AG, Munich, Germany) at 120 kV. A 6 MV radiation beam with 20 × 20 cm^2^ field size, 90° gantry rotation, 85 cm source-surface distance and 250 monitor units was set up and calculated in Eclipse v13.7 (Varian Medical System, Palo Alto, CA, USA) with the Acuros dose calculation algorithm, calculating dose to medium. The calculated dose distribution can be seen in Figure 4 for all bones and implants at 5 cm immersion depth. The same beam was delivered to the water phantom for each implant and setup while the dose was measured with films. Native measurements in front of the implants were not accomplished due to technically limited feasibility and reproducibility in terms of film positioning. However, this part of the beam is checked on a daily basis in the department of Radiation Oncology and is accurate within 2%. The exposed films were converted to doses using a set of pre-irradiated calibration stripes, the Epson Expression 10000XL A3 flatbed film scanner (Seiko Epson Corporation, Suwa, Japan) and the FilmQA Pro 2016 software (Ashland Inc., Covington, KY, USA). These measured dose distributions and planned dose distributions from Eclipse were exported to Matlab R2014a (The MathWorks Inc., Boston, MA, USA) for evaluation. Each dose distribution was normalized by the peripheral dose outside the bone. Profiles and areas of interest were extracted from all planned and calculated dose distributions and compared.

## 3. Results

**Figure 5:**
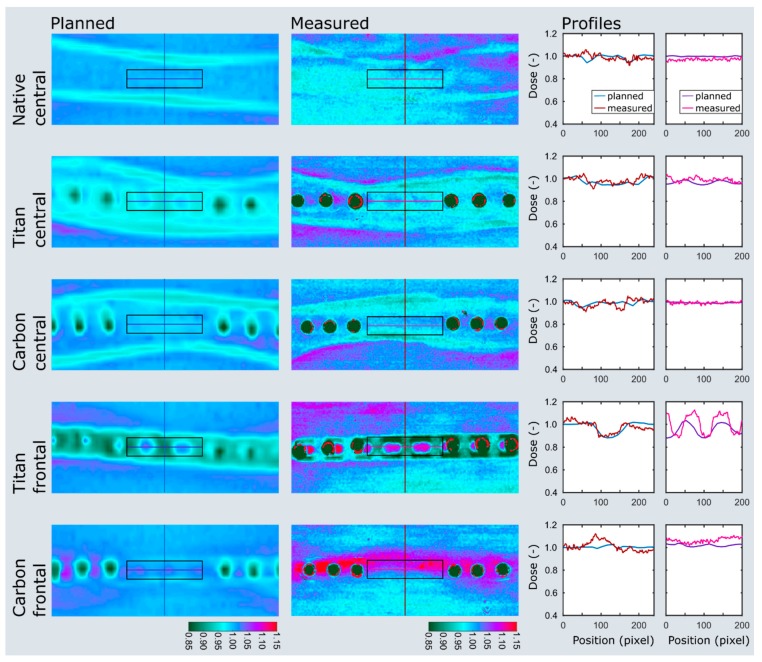
Dose plotting of the plate model at 5 cm immersion depth. The area of interest and profiles are depicted on the planned and measured dose distributions as black boxes and colored lines. Note the distinct dose attenuation at the implant–bone interface with titanium implants (titan frontal) when compared to CF/PEEK implants (carbon frontal). The dose range assimilates in the bone center but remains smaller in the CF/PEEK model (titan central vs. carbon central).

**Figure 6:**
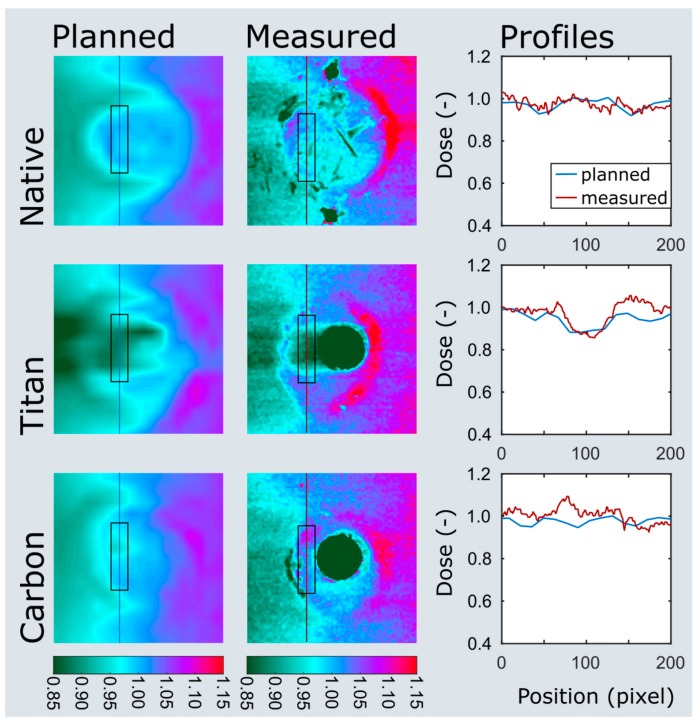
Dose plotting of the nail model at 5 cm immersion depth. The area of interest and profiles are depicted on the planned and measured dose distributions as black boxes and colored lines. Note the clear dose attenuation behind the titanium nail (titan) when compared to the CF/PEEK nail (carbon).

Three pairs of ovine femora were irradiated within a water phantom at two immersion depths. The planned and measured dose distributions of the plate and nail models at 5 cm immersion depth are shown in Figure 5 and Figure 6. Values extracted from the area of interest are listed in Figure S1 in supplementary materials. Both the planned average beam attenuation by the implants (i.e., 1% vs. 5% within the bone in the plate model, 2% vs. 9% in the nail model) and the effective decrease of the mean radiation dosage behind the implants (i.e., 2% vs. 3% within the bone in the plate model, −1% vs. 7% in the nail model) were noticeably weaker when using CF/PEEK implants (Figure S1 in supplementary materials). This effect, however, was even greater at the implant–bone interface and diminished in the bone core (Figure 5).

Particularly in the nail model, the measured radiation dose was in better accordance with the radiation planning using CF/PEEK implants when compared to titanium implants (Figure S1 in supplementary materials, Figure 6). CF/PEEK implants featured various dose increases right behind the implant in all models. The mean dose range associated with CF/PEEK implants was less in the nail model and equal or slightly greater in the plate model than those associated with titanium implants. The radiation dose was not significantly affected by the simulated surrounding soft tissues, as different immersion depths did not reveal a meaningful dose difference in any experimental model. A significant imaging artefact reduction with improved planning feasibility was seen in all CF/PEEK models (Figure 4 and Figure 6).

The planned dose distributions are smoother than the measured ones, as the planned dose distributions are results of a dose calculation on a 3D dose grid with a 1.5 mm resolution, whereas the film measurements (more specifically, the scans of the films) have submillimeter resolution. This results in a smooth calculated dose distribution, but less smooth measured dose distribution, which is more susceptible to measurement and scanning noise.

## 4. Discussion

To the best of our knowledge, this is the first study investigating on the dose-modulating effect of CF/PEEK and titanium implants in long bones in a comparative manner. By means of a cadaveric experimental setup, we found a smaller beam attenuation by CF/PEEK implants, especially when comparing intramedullary nails. This results in a more reliable and more efficient radiation of an osseous target volume. In addition, the dose application was more homogenous with CF/PEEK implants which allows for better radiation planning. In terms of artefact reduction, we endorse the findings observed by Ringel et al. in pedicle screws [5]. The improved image quality might even be the main advantage of CF/PEEK implants in tumor patients, which allows for more accurate diagnostics and facilitates radiation treatment planning.

The most important limitation of this study is a missing baseline measurement in front of the specimens in order to reference the effective dose changes due to the implant and the backscattering by the bone itself. This would have permitted a more precise comparison of the beam attenuating effect of either material as well as a quantification of bone-induced backscattering. However, the fixation of the dosimetry film in front of the specimen and exactly perpendicular to the main beam in a standardized manner was technically difficult and only poorly reproducible. For this reason, the authors decided to refrain from this additional measurement.

The backscattering effect seen with CF/PEEK implants in our series needs to be clearly distinguished to the findings raised by Nevelsky et al. [3]. In their solid water phantom model with Monte Carlo simulations, the maximum overdose due to backscattering was effectively calculated zero for CF/PEEK pedicle screws (both with and without titanium coating). Physically, particles are backscattered at transitions from less dense to denser materials (i.e., from CF/PEEK to bone or from soft tissue to titanium) which leads to a dose increase. However, and most notably, Nevelsky et al. did not analyze the transition from CF/PEEK to bone in their setup but merely investigated the dose change which corresponds to the soft tissue–implant interface in front of the implant. On the contrary, our setup mapped radiation dosage behind the implant and just in front of bone with its higher radiodensity than CF/PEEK. This is why we registered a dose increase mainly in the CF/PEEK specimens. With titanium, the opposite is true with a transition from denser to less dense materials at the implant–bone interface, resulting in a relative dose decrease without backscattering behind the implant.

Comparing the regions of interests for the plate model, a discrepancy of 4–6% between the measured and planned dose at the implant-bone interface was found for CF/PEEK. In our study, this effect is best explained by either imprecision of the film dosimetry or the inaccuracy of the planning system. However, film dosimetry has been well explored and established for mapping radiation dosage with appropriate spatial resolution and should have an imprecision of less than 4%. [12,13,14]. Moreover, the films have been normalized by peripheral reference measurements, which further reduces the risk of false measurements. Ultimately, an inaccuracy of the planning tool with poor modelling of backscattering is the most probable source of this phenomenon. 

Whether or not the presence of titanium screws in the plate model diminishes the beneficial effect of CF/PEEK implants is uncertain. In this regard, it should be borne in mind that the dose-modulating effect of titanium screws within a CF/PEEK plate and distant to the target lesion is still to be determined, and CF/PEEK screws are not readily available on the market. Even though novel radiation algorithms, such as the Acuros or Monte Carlo algorithms deployed in the VMAT (volumetric modulated arc therapy) technique, allow for more homogeneous dose distributions in the presence of metallic artefacts, Ringel et al. could verify beneficial effects of CF/PEEK implants in spinal lesions, in which the VMAT technique is preeminently deployed [5,15]. Therefore, the impact of the implant material on image quality and radiation planning must not be neglected.

Another attempt to explain the greater beam attenuation in the nail model, of course, is the thickness of the implant. The larger the implant diameter to be passed by the radiation beam, the greater the effect of different implant materials. Put in a clinical context, this indicates that patients who undergo an intramedullary stabilization of their radiosensitive metastasis particularly, might benefit from CF/PEEK implants.

From a clinical point of view, plate fixation of metastatic lesions is often supplemented with curettage of the lesion and filling of the defect with acrylic cement [1]. This experimental study, however, did not consider stabilization measures by means of supplementary materials. Nevertheless, bone cement does not cause relevant artefacts and can therefore presumably continue to be used without any concerns in this context.

The clinical significance of these findings cannot, naturally, be demonstrated by our experimental study and should be verified by clinical studies.

This study did solely investigate implants available for the extremities. However, radiation therapy plays no minor role in the treatment of metastases of the spinopelvic region. Future studies therefore should aim at quantifying in situ the dosimetric impact of implants deployed in the axial skeleton.

## 5. Conclusions

In this experimental cadaver study, CF/PEEK implants showed a lesser beam attenuation than equivalent titanium implants. This leads to a more reliable and more effective delivery of radiation dose to an osseous target volume. A considerable reduction of imaging artefacts facilitates radiation planning with CF/PEEK implants. Whether or not these findings are clinically meaningful remains to be investigated. From a radiophysical point of view, the use of CF/PEEK implants is particularly beneficial when intramedullary nails are applied.

## Supplementary Materials

The following are available online at https://www.mdpi.com/1996-1944/13/7/1754/s1, Figure S1: Measured dose deviation with reference to the normalized dose in the periphery. Ti: Titanium; SD: Standard deviation.
