# Supplementary material for: Carbon Fiber/Polyether Ether Ketone (CF/PEEK) Implants Allow for More Effective Radiation in Long Bones
Source: Materials (Basel). 2020 Apr 9;13(7):1754. doi: 10.3390/ma13071754 (PMC7178689; doi:10.3390/ma13071754)
Supplement: Supplementary file 1 [file materials-13-01754-s001.pdf]

materials

MDPI

Supplementary Materials

Carbon Fiber/Polyether Ether Ketone (CF/PEEK) Implants Allow for More Effective Radiation in Long Bones

Christoph J. Laux [1,*,†], Christina Villefort [1,†], Stefanie Ehrbar [2], Lotte Wilke [2], Matthias Guckenberger [2] and Daniel A. Müller [1]

1 Department of Orthopaedics, Balgrist University Hospital, University of Zurich, Forchstrasse 340, 8008 Zurich, Switzerland; villefort@balgrist.ch (C.V.); daniel.mueller@balgrist.ch (D.A.M.)
2 Department of Radiation Oncology, University Hospital Zurich, University of Zurich, Rämistrasse 100, 8091 Zurich, Switzerland; stefanie.ehrbar@usz.ch (S.E.); lotte.wilke@usz.ch (L.W.); matthias.guckenberger@usz.ch (M.G.)
† The first two authors contributed equally to this manuscript.
* Correspondence: christoph.laux@balgrist.ch

Received: 17 February 2020; Accepted: 7 April 2020; Published: date

		NAIL MODEL			PLATE MODEL					
						INTERFACE		CORE		
		Native	Ti	CF/PEEK	Native	Ti	CF/PEEK	Ti	CF/PEEK	
5 CM IMMERSION DEPTH	Mean Dose	0.99	0.91	0.98	1.00	0.89	1.01	0.95	0.99	PLANNED DOSE (ECLIPSE)
	Median Dose	0.99	0.90	0.98	1.00	0.89	1.01	0.95	0.99	
	SD	0.02	0.03	0.01	0.00	0.01	0.01	0.00	0.00	
	D98 (Min dose)	0.95	0.88	0.95	1.00	0.88	0.99	0.94	0.98	
	D2 (Max dose)	1.01	0.97	1.00	1.01	0.92	1.03	0.95	1.00	
	Mean Dose	0.98	0.93	1.01	0.98	0.90	1.05	0.97	0.98	MEASURED DOSE (FILM)
	Median Dose	0.98	0.92	1.01	0.98	0.90	1.06	0.97	0.99	
	SD	0.02	0.05	0.04	0.02	0.01	0.03	0.01	0.02	
	D98 (Min dose)	0.92	0.86	0.89	0.96	0.87	0.99	0.94	0.94	
	D2 (Max dose)	1.03	1.03	1.09	1.02	0.93	1.09	1.00	1.02	
10 CM IMMERSION DEPTH	Mean Dose	0.94	0.93	1.03	0.98	0.93	1.04	0.97	0.98	
	Median Dose	0.93	0.91	1.04	0.99	0.93	1.05	0.97	0.98	
	SD	0.05	0.05	0.03	0.02	0.02	0.03	0.01	0.01	
	D98 (Min dose)	0.86	0.86	0.95	0.95	0.91	0.98	0.95	0.94	
	D2 (Max dose)	1.02	1.02	1.09	1.01	0.97	1.08	0.99	1.00	

Figure S1. Measured dose deviation with reference to the normalized dose in the periphery. Ti: Titanium; SD: Standard deviation.

© 2020 by the authors. Submitted for possible open access publication under the terms and conditions of the Creative Commons Attribution (CC BY) license (http://creativecommons.org/licenses/by/4.0/).
